# Social aspects of living with rheumatoid arthritis: a qualitative descriptive study in Soweto, South Africa – a low resource context

**DOI:** 10.1186/1477-7525-6-54

**Published:** 2008-07-24

**Authors:** Marguerite Schneider, Esther Manabile, Mohammed Tikly

**Affiliations:** 1 Child, Youth, Family and Social Development, Human Sciences Research Council, Pretoria; 2 School of Public Health, Health Sciences Faculty, University of the Witwatersrand, Johannesburg; 3 South African Qualifications Authority, Pretoria, South Africa; 4 Division of Rheumatology, Chris Hani Baragwanath Hospital and University of the Witwatersrand, Johannesburg, South Africa

## Abstract

**Background:**

Rheumatoid Arthritis (RA) is a chronic illness with important functional, social and employment consequences. We therefore undertook a cross-sectional study, using the International Classification of Functioning, Disability and Health framework, to investigate the personal and social consequences of RA in women, living under largely impoverished conditions.

**Methods:**

A qualitative case study design was used with a convenience sample of 60 women with RA living in Soweto, South Africa. Semi-structured in-depth interviews were conducted to cover a range of experiences including onset of disease, treatment, environmental barriers and facilitators, employment, and social inclusion in family and community life. The outcomes are described according the International Classification of Functioning, Health and Disability framework at the body, person and societal levels and looking at both personal and environmental factors.

**Results:**

The main features of living with RA were pain, muscle stiffness at the body level, difficulties in doing various activities such as mobility, washing, dressing, domestic activities, using transport and obtaining and maintaining employment at the person level. At the societal level the participants described difficulties moving around, interacting socially and taking part in community activities, fulfilling social roles and earning a living. Environmental facilitators such as assistive devices and health care services improved functioning. Barriers such as physical environments, lack of transport and basic services, such as electricity, and attitudes of others lead to social exclusion, loss of a sense of self and independence. Low income, lack of sufficient public transport, and sparse basic services were poverty features that exacerbated negative experiences.

**Conclusion:**

The experiences of living with RA in a low resource context are similar to those in mid- and high resource contexts, but are exacerbated by poverty and the lack of basic services. Pain and social exclusion are some of the key experiences of women with RA living in Soweto. The ICF provides a useful framework for describing and understanding the complexity of these experiences.

## Background

Rheumatoid arthritis (RA) is a chronic inflammatory joint disease that is characterised by daily pain, stiffness and fatigue which, in turn, limits activities of daily living. There is increasing awareness that clinical and laboratory markers do not capture the full experience of disability resulting from joint inflammation and deformities with RA. This has led to the need for research that goes beyond the clinical measures to investigate the socio-economic consequences of living with RA. Reduction in the ability to work and concomitant loss of income, an increased need for rest during the day, reduction in leisure activity, difficulties with using transport, additional housing needs and increasing need for social support are some of the socio-economic consequences of RA [1]. Other studies have highlighted how pain [2-4], difficulties with physical activities especially those requiring fine movements such as sewing [3-5], social isolation and loss of self esteem [6,2] and loss of intimate relationships [7] in RA preclude patients from living a fulfilled life.

Significant country and socio-economic context differences in living with RA have been reported. Brekke et al [[8], p1743], in a Norwegian study of people with RA living in two socio-economically different areas with equivalent disease with respect to disease process and joint damage measures, found that those from lower socio-economic areas indicated having worse health and 'also showed less confidence in their ability to influence the disease'. A further study comparing RA patients from Norway and Lithuania showed similar differences in employment, disease activity, physical function, and self reported health status [9]. There is little published work on the social consequences of RA in low-medium income countries. In one study by Mody et al [5] in Kwa-Zulu Natal, South Africa, of mainly indigent Indian and Black South Africans with RA, pain, stiffness and financial difficulties were identified as the main problems.

**Figure 1:**
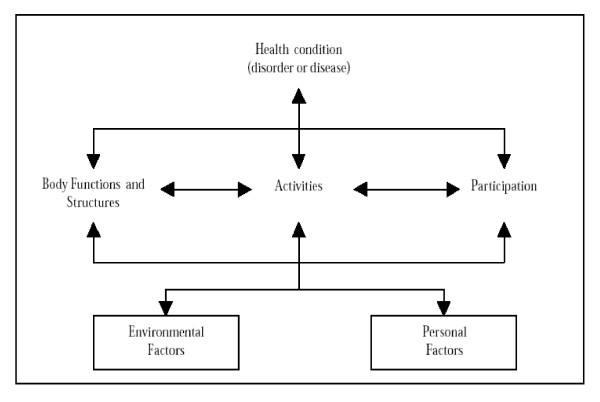
Diagrammatic representation of the ICF framework.

Describing and measuring the consequences of living with a chronic illness falls into the domain of functioning and disability. While there are a number of ways of describing disability, the International Classification of Functioning, Disability and Health (ICF) [10], provides one of the more comprehensive frameworks available to describe the complex and multidimensional phenomenon of disability. The definition of disability, based on the ICF, proposed by the Measuring Health and Disability in Europe Consortium [11] is 'difficulty in functioning at the body, person, or societal levels, in one or more life domains, as experienced by an individual with a health condition in interaction with contextual factors.' The ICF requires one to describe the outcomes at body, person and societal levels and the environmental factors (physical, social/attitudinal and legislative/policy) that act as barriers or facilitators. The theory of disability encapsulated in the ICF is that of the biopsychosocial model where disability is determined by both individual as well as environmental factors. The locus of intervention should, therefore, not be only the individual but also the environment. Figure 1 is a schematic representation of the ICF framework and definition of disability.

We therefore undertook a cross-sectional study, using the ICF framework, to investigate the personal and social consequences of RA in women, living under largely impoverished conditions.

## Participants and methods

A qualitative case study design was applied to a convenience sample of 60 women, fulfilling the 1987 American College of Rheumatology classification criteria for RA [12], and attending the Arthritis Clinic at Chris Hani Baragwanath Hospital, and living in Soweto, South Africa. Soweto is a large township established as a dormitory town to house Blacks during the years of apartheid in South Africa. The sample size was larger than what would commonly be used in qualitative research. This was done to ensure a large enough sample for a standard questionnaire administered during the same interviews. These quantitative data are not reported in this paper as they relate to a different research question.

The interviews comprised a semi-structured, open-ended discussion on the following topics: Time of onset of problem (the actual arthritis and difficulty moving around), the development of the problem and what caused it (as reported by the women), the history of treatment and ongoing support, changes in self image, confidence, employment before and after the onset of the arthritis, general functioning, need and impact of assistance, environmental barriers and facilitators including the physical environment, attitudes of others and services, time use on a typical day, costs associated with having arthritis and income sources available, social relations and social involvement and feelings about quality of life and satisfaction. The participants were also asked to compare good and bad days. The full discussion guide is presented in Appendix A.

Interviews were conducted by one of two research assistants, using a combination of English and interviewee mother tongue (primarily Zulu or Sotho). Questions were not formally translated but, prior to interviewing, a discussion was held between the first author and the research assistants on the different terms to be used to reflect the question content and intent. Interviews took between 30 and 60 minutes and were tape recorded, translated and transcribed into English. Interviews were stopped once 60 interviews had been completed as this was felt to be large enough a sample to provide evidence of trends in the experiences of women with RA living in Soweto, South Africa.

The data were transcribed and translated by the research assistant who did the interviews. The transcripts were analyzed using a basic thematic analysis approach with the main topics covered in the interviews as the starting points. Generally, trends that were reported by the majority of the participants are presented in the results section. The analysis was discussed by the authors and followed a structured analysis looking for responses to the questions asked in the semi-structured interview. Trends and further themes noted within these parameters are reported in the results.

The results are presented within an ICF [10] framework to describe the difficulties experienced by women living with RA. The results are divided according to the level of outcome (Body, Person and Societal levels) described in the ICF as well as the environmental barriers and facilitators relevant for each level. This allows the reader to see the interaction of RA with different environmental factors. In addition, participants' sense of self, satisfaction with life, and social inclusion are described. The latter are not classified or described in the ICF. Direct quotes from the participants are provided as illustrations of these major trends. These quotes have been only minimally edited to retain the flavour of what they said but make their comments understandable to the reader.

The study was approved by the Committee on Research with Human Subjects (Medical) of the University of the Witwatersrand and each participant signed a consent form prior to the interview starting.

## Results

The mean age of the participants was 52.8 yrs (range: 29–60) and they had on average completed 9 yrs of schooling (range: 3–12). Seventeen were married, two were living with a partner, 18 had never been married and 23 were divorced or widowed at the time of interview. Only 13 lived in households without children, while 22 women lived with one child only, eight with two and 17 with more than two children. Eight women lived with no other adults, 11 with one adult, 23 with two to three adults and 18 with three or more other adults.

### The health condition and impairments (body level)

The health condition is RA and the body level impairments relate to individual body parts, systems or organs [10]. The domains discussed here include the onset and management of RA as well as pain as an impairment of body function. The main environmental factors that apply to the body level are health care services and the outcome would be in terms of body level functioning. For example, the provision of medication to control the progression of RA and manage pain.

#### a) Onset and management of RA

The nature and disease duration varied greatly between participants. The disease duration was 5 – 10 years in 43%, 1 – 4 years in 23%, 11 – 25 years in 27% and only for 7% of women was it 26 years or more. Some participants described a more sudden onset and others a more gradual onset, with many participants ignoring the initial symptoms of pain and swelling, and only taking them seriously when they were unable to move and do their daily activities. They all reported significant improvement in their overall functioning as a result of attending the Arthritis Clinic. They were generally positive about the effects of the medication and information provided at the Clinic. A passing comment made by one of the Arthritis Clinic staff suggests that these women are knowledgeable about RA and compliant with treatment. Those who seemed to have a good understanding of RA and attended 'Arthritis school' (a weekly class run by the clinic staff and providing information on RA and coping mechanisms), reported that they had come to terms with the illness, had accepted their state of health, and appeared to be positive despite some difficulties.

#### b) Pain

Most of the participants who responded as having severe and extreme pain, rated their health status as poor and had low future expectations, as evident in this quote.

*Everything I can't do. I can't bath myself without pains and dress without pains, wearing shoes – I can't – they always have to assist me to put on my shoes*.

Most felt that, because pain compromised their health status, their quality of life was also compromised. In cases where pain was the main problem, many reported that they had to leave their employment and, therefore, lost their income and reduced their standard of living. The participants reported pain management as being an important focus for intervention.

#### c) Intermittent nature of RA – good and bad days

Most participants described a bad day as one filled with pain, stiffness and feeling cold. A good day for participants was reported to be a hot sunny day when they had little difficulties (with low pain and stiffness). They complained of not being able to plan in advance and to organise their use of time. It also impacted on how others saw them – sometimes functioning fine and other times unable to perform simple chores. They felt that this lead to others not believing that they were ill.

### Barriers and facilitators in the environment affecting body level functioning

The participants had access to physiotherapy and occupational therapy services for exercises and provision of assistive or adapted devices. This, coupled with attending Arthritis Clinic on a regular basis and being provided with free health care and medication, ensured that the participants all experienced as little pain and joint stiffness as possible. The role of the weather in exacerbating or relieving symptoms of pain and stiffness was a further environmental factor related to the natural environment.

### Activity and participation (person and societal levels of functioning)

This section reports on those activities that a person needs and/or wants to do on a regular or daily basis. The environmental factors related to these activities are discussed with the relevant domains and related quotes, and further discussed at the end of this section.

#### a) Mobility

All participants reported having difficulty in moving around, but the severity of the symptoms varied from day to day and depended on the effectiveness of the medication, the weather and the locus of disease activity (e.g. hands, feet, knees, hips or lower back). Most participants reported difficulties in walking, kneeling, standing, washing clothes or carrying things, and manipulating small objects such as fastening buttons or sewing.

#### b) Self care and domestic chores

The main self care problems experienced by participants were in relation to washing, dressing and putting on shoes. Most needed support from relatives or neighbours and assistive devices to cope with these activities of daily living. Most participants reported having house helpers or washing machines to assist with cooking and washing their laundry:

'*I cannot cook and do washing like I used and my boys are just boys, they do not wash our clothes very clean, but they do their best, and I try to be very understanding when it comes to that.'*

#### c) Commuting and use of transport

Most of the participants reported having to use a minibus taxi to get to places to where they used to walk. These privately owned taxis act as the *de facto *public transport system in Soweto. However, many participants reported difficulties (barriers) in using this service, including the negative attitudes of many taxi drivers towards their disability and difficulties experienced in getting in and out of the taxis. However, many viewed the minibus taxi as an important facilitating service that allowed them to get around, and, in some cases, taxi marshals helped guard their groceries and other wares until they (the women) could send someone to carry the parcels home.

#### d) Use of time

An important correlate of difficulties the participants had in functioning was that of time use. In general, participants reported having to take breaks in between chores, pace themselves, plan ahead for the next day and prioritize on daily activities to balance their workload. Participants consistently reported on how they take longer doing activities since the onset of RA:

'*I wake up at 3:30 am and then warm water. I know by 4 am I will be starting to wash myself and around 6:30 am I'll be finished and go out. I have to wake up very early. If I wake up at around 7 am and have to leave the house at 8 am, I can't go because I'm slow.'*

Because of the intermittent and unpredictable nature of the illness, it was sometimes hard for the participants to plan ahead for the following day. Their plans were often derailed when they were faced with an inability to move on waking up and had to stay in bed. The most significant environmental effect reported here was the need to get up early to warm water because of not having a hot water geyser.

### Environmental barriers and facilitators

#### a) Mobility, self care and domestic work

Access to hot water, an accessible dwelling and other buildings in the neighbourhood, as well as flat, smooth roads were all important environmental factors interacting with the mobility difficulties. Many participants had no access to electricity, running water, or indoor bathrooms and toilets – all significant environmental barriers. Having a hot water geyser and the ability to pay for electricity was viewed as a potential facilitator but could not be afforded by many of the participants.

Common environmental facilitators reported by participants included assistive devices mainly provided by the occupational therapists, such as sponges with long handles to wash their backs, 'push-pull' tap handles, adapted combs and toothbrushes providing a better grip. Some used gloves to wash clothes and dishes to protect their hands from direct contact with water. Interestingly, having a wall to lean on when queuing for services was viewed as a facilitator. Some participants demonstrated creativity in finding solutions. For example, one woman places her clean wet washing in an empty crate with holes underneath to drain the water. Consequently she avoided twisting and squeezing the washing, an activity that causes pain. Others had their washing lines lowered to the level where they did not have to stretch their arms too high. Household gadgets like washing machines, vacuum cleaners and mops were also mentioned as facilitating their everyday life.

Geographical factors such as distance to the shops, crossing the bridge leading to the hospital, and steep unpaved pathways hindered their ability to walk without pain and effort. In order to avoid shorter but rocky pathways, many participants opted to use longer but more even-surfaced pathways.

#### b) Additional costs

The main additional costs incurred included increased use of taxis or special transport, paying others to do the cleaning and washing, visiting traditional healers or buying additional over-the-counter medications. Indirect costs included loss of or reduction in income compared to before the onset of the illness. Special weight reduction diets were another source of additional costs, where participants had to buy food that was different to what the rest of the family ate. Most of the participants depended on the monthly state social security benefit for disability (disability grant), which, for many, was much less than their income when still working.

#### c) Attitudes of others

The attitudes of others, both within the family and beyond played a significant role in determining participants' experiences. Within their close family, participants commonly felt accepted, understood and supported and expressed this as an important facilitator. Only a few reported not being close to their families and felt RA had driven them away from their families. In general, they felt that strangers were less understanding.

'*I avoid walking on the streets. I rather stay indoors or stay with my family. I feel angry because I know inside they say 'ag, shame, I wonder what is happening to her', those who don't know me. But those who know me will say, 'it's arthritis'.'*

The negative attitudes of others also manifested itself in the disbelief in the participants' illness due to their inability to do things one day and being able to do the same activity the following day. This is the intermittent nature of RA.

Not many participants talked about their intimate relationships. Those who did, however, expressed strong feelings of not wanting to be in a relationship. The reasons given included the physical pain causing discomfort in 'having another body in bed' or even just being touched. One participant said things were a bit rocky in her relationship with her husband as he was not supportive and struggled to understand her short temper and impatience. Others also explained that their partners had become 'bored' and did not tolerate their moods. A number of participants were in fact divorced, and gave having RA as a significant contributing factor to their divorce. Thus, the lack of support and attitudes of spouses were seen as one of the barriers to maintaining intimate relationships.

#### d) Employment opportunities

Most participants were unemployed, some being forced to leave their employment because they could no longer manage the pressures of work. Others still worked but moved to a less demanding positions and with a lower salary. The nature of the job opportunities available to these women living in a low resource context were generally ones that were labour intensive in terms of physical activity, such as domestic or factory work, nursing or self employment in small business opportunities.

'*I used to cook and sell soft porridge at the pay stations for pensioners. I would get up at 4 am and cook then push my wheelbarrow with buckets full of soft porridge and walk around and the pay station selling to pensioners in a tray with mugs. Now I can't even carry a 5-litre, so I stress a lot when I think about it.'*

### Personal factors

Some participants accepted that the problem might not be with other people only but that they themselves were the ones who had changed since the onset of RA. Several participants reported feeling unworthy and looking down upon themselves.

'*I do not keep in touch with them (friends), since I got sick, I feel unworthy and look down upon myself and feel I do not belong with them*'.

Other participants felt they were a burden to people, and avoided mixing with people in community events and visiting families. Many of them, thus, remained within their close knit family context or with close friends. Some reported staying close and feeling comfortable with others who have the same health problem or other chronic health problems, therefore forming informal support groups.

'*My friends stay on the same street and they have got arthritis and the other one has got diabetes. So I visit them with no problem and we help each other out.'*

Some participants reported that they have stopped visiting family and friends in part because of the difficulty in getting around and in part because of not wanting to be confronted with negative attitudes of others. Some felt that even their own relatives had negative attitudes towards them because of not being as productive as before, for example, in helping cook for a funeral or family gatherings.

### Independence, self confidence and self identity

This section highlights the overarching impacts of living with RA. These are all complex and beyond the ICF but remain important outcomes of the experience of living with a chronic illness.

#### a) Independence

Independence was a strong theme running through all the interviews. Participants expressed feelings of sorrow and distress at not being able to do things for themselves and having to rely on others. This loss of independence angered many of the participants, as they had lost a sense of control and felt that they were too dependent on others.

'* [The arthritis] changed my life. I saw myself not being able to do anything for myself.....You know, sometimes even when I see that I can't do something, I force myself to do it because I don't want to bother other people. I do things myself. This feels bad because I feel I am bothering people.'*

#### b) Self confidence and acceptance

Most participants reported having lost their sense of confidence in part due to their dependence on others and in part because of fear of what others will say about them. Some had learnt to accept RA and attend 'Arthritis school' to learn how to manage their pain and cope in their daily living, whereas some had just given up hope and allowing the disease to 'rule their lives'.

Some participants hid their deformed bodies from friends and other people but felt comfortable when they are at the clinic for treatment when meeting people with the same problems.

'*I used to hide my arms so that people wouldn't see until I met with other people with arthritis, then I accepted who I was and the way I was*'.

Only a few participants reported still having a positive approach towards life and believing in themselves, especially when it came to participating in usual activities. Some participants reported an acceptance of their way of life and health status and were trying to make the best out of a bad situation. Others were less accepting and felt they still needed more out of life and, because of RA, would not achieve this.

'*I am not satisfied with my life today because I cannot even find a job for myself to take care of my family.'*

#### c) Self identity

Identity is a complex field and includes a person's sense of self developed through one's biography. For participants in this study the main identity issue was about differentiating themselves 'before' and 'after' onset of their symptoms. When participants were asked to self identify as being disabled or not, about half identified themselves as being 'not disabled', a few identified themselves as being 'disabled', and the remainder indicated a hesitancy to identify themselves as disabled but acknowledged that others would identify them as disabled. The reasons given for identifying themselves as disabled or otherwise varied. Some believed that disability as an identity was fluid and changed over time, depending on the level of pain and difficulties experienced in doing activities. For others, identifying as disabled depended on what others saw, defining disability as a physical observable body deformation. Participants who did not show signs of deformity tended to report themselves as 'not disabled'. Independence and the ability to do things for oneself were seen as crucial factors in self identification as disabled or not.

## Discussion

The impetus for the study was a desire to develop a better understanding of the lived experience of RA in an urban South African community – thus providing greater insight into outcomes of clinical interventions 'beyond the clinic'. In addition, the study provides some evidence on the additional impact of a low resource context on the experience of living with RA. The major factors reported by the participants as determining their experiences are pain, social exclusion and loss of independence and sense of self worth.

For most participants, pain and, to a lesser extent functional disability, had a widespread impact on relationships, psychological well-being, ability to work and recreation. Pain is invisible and hard to measure and, yet for participants, there seemed to be a correlation between level of pain and self perceived health status with pain negatively influencing perceptions of life quality. Previous studies in RA have shown that pain is a major factor in determining quality of life as well as being the single most important symptom that sufferers want effectively managed [3,13]. In the present study, the effect of pain on functioning was reported by the participants as limiting their participation in social life leaving them feeling isolated, depressed and frustrated. The intermittent nature of pain was poorly understood by outsiders who saw a person able to do an activity one day and not the next. Moreover, the intermittent and unpredictable nature of the pain severely impacted on the participants' ability to organise and plan their time. Many had stopped working because of pain. Finally, in order to reduce pain levels, many participants spent scarce financial resources to buy over-the-counter analgesics.

While the study did not have a specific measure of social exclusion, there was a common recurring theme on the limited social interactions. Social exclusion was imposed both by the negative attitudes of the community (environmental factor) and personal factors affecting participants, such as a reluctance to go out and socialise because of difficulties with mobility and use of transport, and negative self-perception. The resulting isolation made the participants, to some extent, feel safe as it limited their need to face the outside world. But this isolation also limited their contact with the outside world and further reduced their social interactions and the roles that they play in their family and community. An example is the common comment made by the participants about not being invited to family gatherings and not being able to contribute to the cooking required at these events. Conversely, those with strong support structures were able to remain more socially active. Attending weddings, funerals and community gatherings are important social activities in areas such as Soweto with women playing an important role in cooking and organising these events.

Many participants experienced a strong difference between their sense of self before and after the onset of RA. Role shifting was a common occurrence in relation to the loss of independence and having to rely on others to assist and lose their role as breadwinner and caregiver. Some of the important roles of women in the context of Soweto, and many other Black South African settings, are care giving (often of grandchildren) and providing food (e.g. cooking at gatherings) [14]. The inability to fulfil these roles by many of the participants in the study seemed to be an important loss in their sense of self and being part of the community.

These findings reflect similar findings for other chronic illnesses. Ahlström [[15]; p79] describes the experiences of loss and sorrow of people with chronic illnesses. These are categorised into loss of 'bodily functions' (e.g. walking, strength), 'relationships' (e.g. spouse, friends, community), 'autonomous life' (e.g. independence, self-determination), 'life imagined' (e.g. dreams, being healthy), 'roles' (e.g. family and occupational roles), 'activities' (e.g. work, leisure pursuits), 'identity' (e.g. worth, self-confidence), and of 'uplifting emotions' (e.g. hope, relish for life). Other studies report loss of control over social participation because of the unpredictability of multiple sclerosis [16], loss of independence [17], psychological adjustment to chronic illness and disability [18], and the protective factors of social support, individual personality traits and spiritual beliefs [19]. Misajon, Manderson, Pallant, Omar, Bennett & Rahim [20] noted that the most significant impact and distress experienced by people with a physical impairment is not in the realm of self care and physical activity, but rather in those of social interactions and social roles.

Negative external environmental factors, especially those consequent of low resource context such as the difficult physical environments with respect to roads and transport, lack of basic services such as electricity and hot water geysers, and lack of appropriate job opportunities created significant barriers for the participants. While these were not highlighted by the women as the main problems they face, these environmental barriers contribute to and compound the problems of social exclusion, loss of self esteem and self identity as a contributing member of their households and broader society. By contrast, the availability of good health care services at limited cost to the participants and the disability grant (a state social security benefit) were perceived as strong facilitators. In addition, the employment opportunities available to the women tended to be ones requiring low skills, high levels of physical activity and generally with low salaries. The onset of RA made it difficult for the women to maintain these employment opportunities because of their difficulties. Furthermore, attitudes of employers in these workplaces would not have been particularly sympathetic to making the required accommodations for the participants in the current climate of high unemployment where finding a replacement low skilled worker is easy. Despite the many environmental barriers the resilience of the participants came to light in their various solutions to difficulties they experience; for example, using the crate with holes to drain washing and asking taxi marshals to look after their shopping bags until they can send someone to pick these up.

While other studies have highlighted the role of socioeconomic context on health status and quality of life, these studies have not looked at the specific role of different factors within these contexts that contribute significantly to these findings [8,9]. The current study provides some evidence of specific aspects of a low resource environment which contribute negatively or positively to health status and quality of life. Further research is required to understand these effects more comprehensively. The resilience described above, however, is one example of these women finding cheap, home made solutions to difficulties they face as the services to provide solutions are generally not available.

The ICF highlights the complex relations between different components in disability and, as such, provides a useful framework to explain and understand the experiences of living with RA. The experiences of the women were a clear outcome of the interaction of their RA with the context in which they live – the physical structure of buildings and the geography, social support and attitudes and services provided (basic municipal services, health care and transport). The outcomes were at the body level (pain, joint stiffness), person level (difficulty moving around, doing fine hand movements, self care, obtaining and maintaining employment, etc.), and societal level (loss of employment, difficulty in moving around because of the geography and poor public transport, loss of social interactions because of negative attitudes, etc.). The participants also provided many examples of environmental barriers and facilitators that include assistive devices, the built environment, the natural environment, support and attitudes of others, and services and policies.

The descriptions provided by the participants support the multidimensional and complex nature of disability embodied in the ICF model. For example, the difficulties experienced in moving around and the intermittent nature of these difficulties lead to attitudinal barriers from others. These negative attitudes in turn lead to the women not wanting to go out and becoming socially isolated and feeling depressed (as reported by the women and not formally assessed) – a body level impairment of emotional function. Another example is the lack of basic services such as adequate public transport and easy access to electricity for warm water which further complicated the women's lives, such as having to get up very early to warm water or pay for transport and experience negative attitudes of taxi drivers. The description of these experiences using the ICF allows for a better understanding of the determinants of people's life satisfaction, level of independence, self-confidence and identity.

Some of limitations of the study include the cross-sectional nature of the study done in a tertiary care setting and not categorising participant responses with respect to disease activity and severity at the time of the interview. As alluded to earlier, disease activity fluctuates and this might impact on the responses of participants. Furthermore, undertaking observations of the women in their daily activities would provide further information to triangulate the description of their experiences of living with RA.

## Conclusion

The participants' description of their difficulties and living with RA provides evidence of the complex interaction of a person with a health condition and the context in which they live. The similarity of the experiences of the women from Soweto to those of women in Europe also living with RA highlight the universal nature of the effect of this condition on people's lives. However, there are also important differences between these two contexts, where women in Soweto seem to experience an additional burden of poverty in the lack of services and low income levels. This reduces the support that they are able to harness to cope effectively.

Notwithstanding the limitations of the study, we believe our findings provide important insights into social consequences of RA in a low resource context. The study highlights the paramount need for pain control and measures to reduce environmental barriers and increase facilitators for patients with RA. The latter not only requires improvements in public amenities and utilities such as public transport and electricity, but also education of the wider society and employers about the nature of RA and the potential benefits of a progressive attitude for RA patients and their families.

Further research on the consequences of living with RA should look at the effect of interventions focused on different environmental factors to determine the cost effectiveness of various interventions. This research could include, for example, investigating the relative benefits of providing only appropriate medical and rehabilitation services compared to also ensuring adequate access to basic services and transport on overall functioning and quality of life. The ICF framework provides a means to describe people's level of functioning to allow a comparison between functioning and satisfaction with and subjective rating of quality of life. Similar studies for different health conditions would add to the growing body of evidence on the social aspects of living with a chronic or other illness.

## Competing interests

None of the authors have any financial or other competing interests. The study was partially funded by the Connective Tissue Research Fund of the University of the Witwatersrand.

## Authors' contributions

MS and MT designed the study and took responsibility for the final write up of the study. EM did the data collection and initial analysis together with MS. All authors read and approved the final manuscript.

## Appendix A: Discussion guide for the semi-structured interviews

IN-DEPTH INTERVIEW QUESTION GUIDELINES : topics to be covered and some probe questions

1. Time of onset of problem (arthritis and difficulty moving around), story of the development of the problem and what caused it

• How long have you had this problem with movement?

• Tell me the story of how it happened.

2. History of treatment and ongoing support

• Tell what happened in terms of treatment you received and other support

3. Changes in self image and confidence : comparing before and after the onset of the arthritis

• Tell me about the person you were before this happened.

• Tell me about the person you are now.

• What are the main things that have changed for you because of the arthritis?

4. Changes in education and/or employment : comparing before and after the onset of the arthritis

• Were you working or studying before the onset and how did that change?

• Are you working or studying now?

• If not, would you like to be? And what is stopping you?

5. General functioning and aspects where assistance is needed

• You have told me quite a lot about what you can do and where you have difficulty. Could you tell me very briefly what is the main area in which you have difficulty (i.e. not able to do for yourself)? And how do you feel about that?

• What things are very important to you and that you able to do for yourself? How do you feel about that?

6. Environmental barriers and facilitators : physical environment, attitudes of others

• Think about what things you have at home or other places that make it easier for you to do different activities. What are these? (e.g. sponge on stick to wash yourself, warm water, adapted toothbrush or hairbrush, etc.)

• What about getting to the shops or to the clinic? What makes it easier?

• Now think of the things that make it difficult at home or in other places. What are these? (e.g. stony pathways, too many steps, no hot water, etc.)

• What about attitudes of other people towards you because of the arthritis – has this been an issue for you? And have these been positive or negative, understanding or not understanding?

7. Time use – what person does on a typical day

• Tell me about your day – just an ordinary, typical day. Describe for me what you do and how long it takes you to do things.

• How different is this to a typical day before you had arthritis? Specifically tell me how long you took to do things compared to now. (e.g. getting dressed, washing yourself, getting to the shops, etc.)

8. Cost of having arthritis and income sources

• Tell me about the extra costs you have because of the arthritis? E.g. medicines, additional transport, paying people to assist you, etc.)

• How do you manage to pay these extra costs?

• Do you get a disability grant or old age pension from the government?

9. Social relations and social involvement – attending community meetings, stokvel, church groups, family gatherings, having friends, etc. : comparing before and after the onset of arthritis

• Tell me about the other people in your life today – the types of relationships you have, etc. Has this changed since the arthritis started?

• Do you belong to any organisation or groups (e.g. church groups, choir, stokvel, etc.) ? Has this changed since the arthritis began?

10. Feelings about life – quality of life and satisfaction

• What makes your life happy?

• What makes your life difficult?

• How satisfied are you with your life today?

11. If I had to ask you 'Are you disabled?' what would you answer?
